# Beyond sightings: Ensemble modelling to identify conservation hotspots of Smooth-coated otters *(Lutrogale perspicillata)* in the Ganga River Basin for informed policy and sustainability

**DOI:** 10.1371/journal.pone.0353661

**Published:** 2026-08-19

**Authors:** Syed Ainul Hussain, Ashish Kumar Panda, Surya Prasad Sharma, Naureen Shahniaz, Shivani Barthwal, Sk. Zeeshan Ali, Ruchi Badola

**Affiliations:** 1 Ganga Aqualife Conservation and Monitoring Centre, Wildlife Institute of India, Dehradun, Uttarakhand, India; 2 Centre for Biodiversity and Sustainability Science, Doon Officers Enclave, Dehradun, Uttarakhand, India; Asian Disaster Preparedness Center, THAILAND

## Abstract

Among the three species of otters found in India, viz. the Smooth-coated otter (*Lutrogale perspicillata*), Eurasian otter (*Lutra lutra*), and the Asian small-clawed otter (*Aonyx cinereus*), the Smooth-coated otter has the widest distribution. Historically, the Himalayan foothills and the Gangetic floodplain constituted the stronghold of Smooth-coated otters; however, changing land uses to accommodate growing human populations have led to extensive habitat loss. Despite its ecological importance as a top predator and indicator of river health, systematic, basin-scale assessments of Smooth-coated otter habitat remain limited. In the Ganga River Basin (GRB), available occurrence records are sparse, spatially fragmented, and largely opportunistic, constraining the identification of priority habitats and the formulation of effective, evidence-based conservation strategies. To address this knowledge gap, we undertook comprehensive boat-based visual encounter surveys and complemented by secondary occurrence data to evaluate habitat suitability for otters across the GRB. We surveyed approximately 7,680 km of the river stretches across 22 tributaries, including the mainstem Ganga, between 2016 and 2024. We performed habitat suitability using Ensemble modelling, incorporating 26 predictor variables. The model yields high predicted accuracy, with an AUC value of 0.91 ± 0.03 and a TSS of 0.71 ± 0.06. Distance to Protected Areas (PAs) and distance to rivers emerged as the top predictor variables that highly influenced the habitat suitability for the Smooth-coated otter. Habitat suitability indicated that only 0.26% of the area constitutes highly suitable habitats for the species in the GRB. Overall, our findings provide key insights for conservation planners and managers for developing otter conservation strategies in the Gangetic floodplain of India.

## Introduction

In the Anthropocene, human activities have spread to nearly all corners of the Earth, almost overrunning all ecosystems, including those that were previously untouched [1], particularly freshwater ecosystems [2]. Consequently, the conservation of freshwater species became challenging, as they inhabit dendritic and linear habitats that are more vulnerable to threats like habitat fragmentation, loss of connectivity, and flow alteration [3]. These threats, acting alone or synergistically, lead to geographical isolation and limit gene flow, and ultimately increase the risk of local extinctions [4–10]. Additional threats, including invasive species, pollution, and climate change, further threaten the freshwater ecosystems and species [11–13]. Since 1970, global biodiversity has declined by more than 80%, a rate twice as rapid as observed in marine or terrestrial ecosystems [14–16]. Despite the evident decline, freshwater biodiversity continues to receive limited attention in conservation legislation and policy frameworks [11,17–19].

India exemplifies these global challenges with the seventh largest geographical area of around 3.6 million km2. The country supports a vast network of freshwater habitats, including rivers, ponds, lakes, floodplains, groundwater, wetlands, streams, cave waters, marshes, bogs, and swamps [4,20–22]. These freshwater systems provide critical ecological, social, and economic values, yet they are increasingly threatened by pollution, habitat loss, over-exploitation, and climate-change driven hydrological changes [23–26]. As the scarcity and competition for freshwater increase with the growing human population, it has become vital to understand the way such pressures affect the habitat availability and suitability for freshwater biodiversity.

The Ganga River Basin (GRB), one of the largest river basins in South Asia, contributes substantially to India’s freshwater resources and continues to function as a stronghold for several aquatic macrofauna, including the Critically Endangered Gharial (*Gavialis gangeticus*), the Vulnerable Mugger crocodile (*Crocodylus palustris*), the Endangered Gangetic dolphin (*Platanista gangetica*), several freshwater turtle species, and otters [7,27,28]. Three of the otter species, namely the Eurasian otter (*Lutra lutra*), Asian small-clawed otter (*Aonyx cinereus*), and Smooth-coated otter (*Lutrogale perspicillata*), are found in India, particularly the Ganga River Basin [29].

The Smooth-coated otter (*Lutrogale perspicillata*) exhibits the broadest distribution among Indian otter species, ranging southward from the Himalayan foothills and even into the arid zones of western India [30,31]. It inhabits a diverse array of aquatic ecosystems, including forested rivers, freshwater wetlands, reservoirs, lakes, and coastal mangrove systems [32–34]. It has also been recorded to inhabit irrigation canals and rice fields near human civilizations [35–37]. Primarily piscivorous, the species depends heavily on aquatic environments for sustenance, although it also preys on birds, amphibians, insects, crustaceans, small mammals, and reptiles when available [30,31,36,38–40].

Despite its extensive geographic range, the Smooth-coated otter is experiencing severe declines across its distribution range [41,42]. The species is currently classified as Vulnerable on the IUCN Red List due to threats such as habitat degradation and loss, falling prey populations, pollution, poaching, and illegal trade [36,38,43]. The species is a freshwater habitat specialist that depends upon extensive and well-connected stretches of riparian habitats to support a viable population, which makes it more at risk to conservation threats [30]. As the top predator in various food chains, it acts as a bio-indicator of ecosystem health, as its presence in a habitat implies the presence of prey species and good water quality. Since they support the overall resilience of freshwater ecosystems, it is imperative to protect them from further population declines [44–46]. However, in the GRB, recent and systematically collected information on the species distribution and habitat preferences remains limited and spatially fragmented [47–49].

Previous species distribution modelling (SDM) studies of the Smooth-coated Otter across South Asia have provided valuable insights into its potential distribution and environmental associations [38,41,42,50–52]. However, these studies were often constrained by limited spatial extent, coarse environmental predictors, incomplete representation of anthropogenic variables, or insufficient model validation [50,53]. As a result, their capacity to capture basin-scale spatial heterogeneity and the complex ecological gradients characteristic of large river systems remains limited [34,38,54]. Addressing these limitations is essential for generating conservation-relevant predictions in dynamic riverine landscapes such as the Ganga River Basin.

SDM of elusive and wide-ranging species like otters is further challenged by sparse and spatially biased occurrence data, which can lead to overfitting and unreliable predictions when single modelling algorithms are used [34,41]. Ensemble modelling frameworks, which integrate predictions from multiple algorithms, have been shown to reduce individual model biases, improve predictive accuracy, and better quantify uncertainty [55–58]. Advances in SDM methods and computational tools have made it increasingly feasible to predict suitable habitats and assess the relative influence of environmental and anthropogenic drivers, even for species with limited occurrence records [59–61]. Ensemble approaches are particularly effective under such conditions, as they can produce robust predictions from small or unevenly distributed datasets [62–65]. This multi-scale workflow enabled (i) detection of previously surveyed suitable stretches, (ii) identification of river-corridor conservation hotspots clipped from a complete species range model, and (iii) generation of actionable policy-aligned conservation recommendations for India and Nepal [41,62–65].

In this study, we combined species occurrence data from boat-based visual encounter surveys and secondary occurrence records to assess the habitat suitability of the Smooth-coated otter across the Ganga River Basin. The objectives were to (i) (i) evaluate the habitat suitability of the species within the GRB and (ii) quantify the influence of climatic, habitat, and anthropogenic variables on habitat suitability using an ensemble modelling framework. By identifying river stretches that constitute highly suitable habitats, this study aims to support evidence-based prioritization of conservation actions and threat mitigation strategies.

## Methods

### Study area

**Fig 1:**
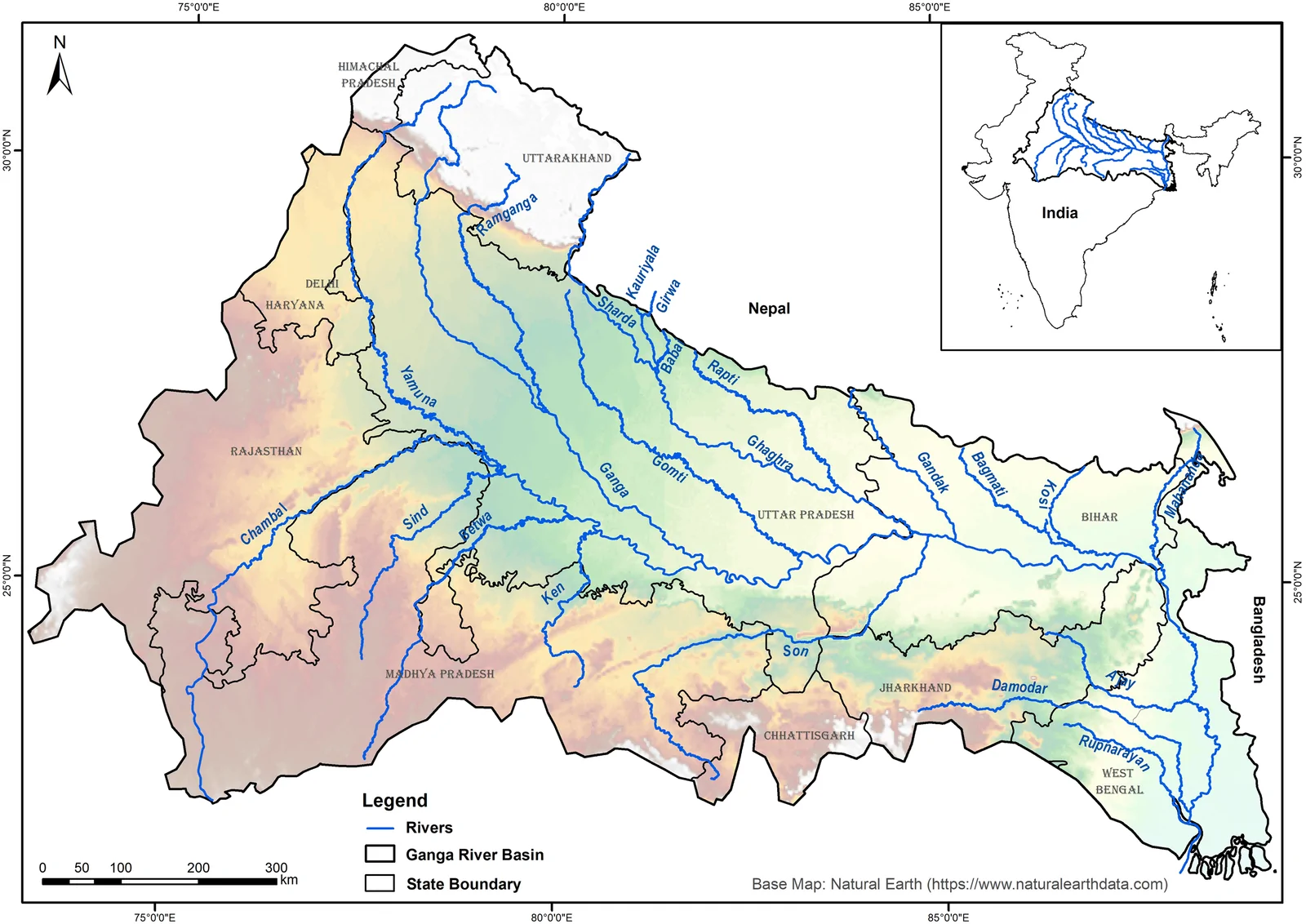
The Ganga River Basin and its 22 tributaries. Base Map: Natural Earth (www.naturalearthdata.com).

The GRB is among the largest river basins globally, covering roughly 1,080,000 km^2^, of which 861,404 km^2^ fall within India [66] (Fig 1). The basin is delineated by the Himalayas to the north, the Chota Nagpur plateau and Vindhyas to the south, the Aravalli range towards the west, and the Brahmaputra Ridge towards the east, leading to considerable climatic, topographical, and hydrological heterogeneity [67]. The snow-fed rivers of the basin, notably the Ganga, Yamuna, Ghaghra, Gandak, and Kosi, sustain a consistent water flow year-round, while rain-fed rivers like Son and Rupnarayan exhibit more pronounced seasonal variations (Fig 1). The Ganga River Basin has one of the densest human populations, which exceeds the national average of 382 individuals per square kilometer for most of the basin, except the state of Uttarakhand [68]. The elevated population density has resulted in increased pollution and the demand for water for agricultural, industrial, and domestic purposes, frequently compromising wetland integrity and diminishing riverine habitat quality. Sand mining, hydro-power projects, and riverfront development have led to habitat fragmentation, diminishing the availability of appropriate resting and breeding sites for otters [69–72]. The climate of the Ganga River Basin ranges from alpine at greater elevations to temperate, subtropical, and tropical at the lower elevations. The average annual precipitation ranges from 500 mm to 2,500 mm, with June through September accounting for roughly 80% of the total rainfall [73].

### Survey methods

In the GRB, a boat-based visual encounter survey was undertaken from 2018 to 2024 to assess the occurrence and habitat use of Smooth-coated otter. 22 rivers encompassing 7,680 km were surveyed (Fig 1). Each surveyed river was divided into 5 km segments, referred to as Biodiversity Evaluation Units (BEU), using ArcGIS 10.2 (ESRI, Redlands, USA). The boat speed was maintained at 6–8 km/h to ensure systematic coverage of the river banks. Each survey team comprised two observers equipped with NIKON 8 × 42 binoculars. All observers focused on identifying otter sightings, including direct sightings, tracks, spraints, grooming sites, and dens along the river banks.

### Data collection and environmental variables

To assess suitable habitats for the Smooth-coated otter, we collected occurrence records from field surveys that were conducted from 2018 to 2024. Additionally, secondary data were obtained through GBIF (www.gbif.org, accessed August 2024), published literature, and reports. A total of 712 presence points (survey = PQ; literature = RS) were gathered. To minimize sampling bias and over-fitting in species distribution models, a spatial filtering approach using the spThin package implemented in R was used to remove duplicate occurrences within a single (1 × 1) km grid cell, resulting in a final dataset of 267 (712 reduced to 267) occurrence points for analysis [74].

A total of 26 environmental variables were used for habitat modelling, including climatic, topographic, habitat, and disturbance factors. Land use and land cover (LULC) data were derived from the Moderate Resolution Imaging Spectroradiometer (MODIS) Land Cover Type (MCD12Q1) at a spatial resolution of 500 m. To evaluate the proximity to types of habitats preferred by the species, Euclidean distances from rivers, roads, and protected areas were determined. Through Google Earth Engine (GEE), data such as Normalized Difference Water Index (NDWI) were derived from MOD09GA surface reflectance data from AppEEARS [64], and then the Annual Mean Normalized Difference Vegetation Index (NDVI) was derived from MODIS. As characteristics of habitat quality, a greater value of NDWI shows the stability in water availability, while a greater value of NDVI represents dense vegetation. Furthermore, data on elevation were derived from WorldClim Version 2.1 [75]. Furthermore, MODIS-derived variables (500 m) were resampled to a uniform 1 × 1 km spatial resolution using mean-aggregation resampling, to ensure spatial consistency across predictors.

The predictor variables were selected based on their relevance to Smooth-coated otter ecology. After testing for multicollinearity using the Variance Inflation Factor (VIF), a threshold of VIF ≥ 10 was applied, and a total of 16 uncorrelated covariates were retained for the SDM (Table 1). The variables were uniformly resampled at 1 × 1 km spatial resolution using Resample tool in ArcGIS to ensure spatial consistency across all variables. The entire extent of the distribution of the Smooth-coated otter was considered for model training to avoid overfitting to local conditions and biased background selection associated with restricted training extents. Also, the Background points were randomly sampled (method = *eRandom*) within the model training extent and treated as pseudo-absence locations for model calibration. This approach follows established guidance on the use of presence-background points as pseudo-absence in species distribution modelling to reduce sampling bias and improve model generalization [76,77].

**Table 1:** Environmental variables used for modelling the distribution of Smooth-coated otter with their VIF values.

Variables	Names	VIF
Mean Diurnal Range	bio2	3.29
Isothermality	bio3	2.35
Mean Temperature of Wettest Quarter	bio8	5.73
Mean Temperature of Driest Quarter	bio9	6.02
Precipitation of Wettest Month	bio13	3.70
Precipitation of Driest Month	bio14	8.04
Precipitation Seasonality	bio15	5.15
Precipitation of Driest Quarter	bio17	8.63
Precipitation of Warmest Quarter	bio18	2.35
Precipitation of Coldest Quarter	bio19	1.73
Distance to Protected Area	EUC_PA	1.36
Distance to River	EUC_RIVER	1.75
Distance to Road	EUC_ROAD	2.61
Human Indices	HII	1.33
Normalized Difference Vegetation Index	NDVI	1.95
Normalized Difference Water Index	NDWI	2.37

### Habitat suitability model building and validation

Habitat suitability models for the Smooth-coated Otter in the Ganga River Basin were developed using an ensemble species distribution modelling framework implemented through the SDM package in R version 4.4.2 [78]. The modelling framework and underlying algorithms follow the SDM implementation described in the SDM package [79] and ensemble design principles outlined for multi-model inference in species distribution studies. We employed niche-based species distribution modelling algorithms, integrating both regression and machine-learning approaches to capture complementary model structures. The candidate algorithms included: (i) Generalized Linear Models (GLM) with second-order polynomial terms, using stepwise predictor selection optimized via Akaike Information Criterion (AIC); (ii) Generalized Additive Models (GAM) with spline-based smoothers to model non-linear ecological responses; (iii) Boosted Regression Trees (BRT) to account for complex interaction effects and hierarchical splits; (iv) Random Forests (RF) with bagged decision trees for robust, non-parametric classification; (v) Multivariate Adaptive Regression Splines (MARS) to model threshold-driven piecewise relationships; and (vi) Maximum Entropy (MaxEnt) modelling for presence-background probability estimation [80–82]. Model calibration was performed on across the distribution extent to ensure unbiased species environment representation, and results were subsequently clipped and classified for the Ganga River Basin [56].

The performance of the model was evaluated through two complementary metrics the True Skill Statistic (TSS) [83] and Area Under the Receiver Operating Characteristic Curve (AUC) [84]. A threshold-dependent metric, TSS, measures sensitivity and specificity. TSS has a value range of −1 to +1, with the higher values indicating a greater accuracy in the model. On the other hand, AUC is a threshold-independent metric that represents the ability to discriminate between the classes by the model. AUC has a value that ranges from 0.5 (no discrimination) to 1 (perfect discrimination), with the higher values indicating a greater performance by the model. A committee averaging approach that retains algorithms with a value of AUC ≥ 0.90 was used to obtain the final ensemble model [85–87]. Pseudo-absences were generated using stratified random sampling outside a 1 km buffer of known occurrence clusters to maintain spatial contrast while avoiding sampling overlap. The final presence–absence threshold was selected at the peak TSS value (0.25), with additional validation via density separation of predicted probabilities.

Predictors were resampled to 1x1 km spatial resolution, and model calibration was performed across the total extent to maximize spatial independence. Background points (n = 1,000) were randomly generated using *eRandom* and treated as pseudo-absences for model training, consistent with Ensemble of Small Models guidance [54]. Model replication used subsampling and bootstrap resampling (n = 60 total models; training/test split = 70/30). Ensemble weighting and consensus thresholds were based on TSS-optimized weighted averaging to support basin-level conservation inference [54].

The relative importance of the variables was assessed to understand the importance of the selected environmental variables. This was done by comparing the extra weight of each factor (Boyce Index) to the total of the weights of all the factors used in the model. For the analysis of the effect of the environmental factors on the habitat suitability of the Smooth-coated otter, response curves were created in ggplot2 (version 3.3.3) [88]. Furthermore, the habitat suitability was classified into three types: low (0.0–0.49), medium (0.5–0.74), and high (0.75–1) based on the probability values. For ease of interpretation and to support conservation prioritization, we merged the high and optimal categories into a single “high-suitability” class.

The study involved non-invasive, boat-based visual encounter surveys and habitat assessments, and did not include any experimental handling, capture, or invasive sampling of live animals. Therefore, in accordance with the Institutional Animal Ethics Committee (IAEC) and national wildlife research guidelines, separate ethical clearance was not required.

## Result

The habitat suitability model for Smooth-coated otter in the Ganga River Basin yielded an AUC of (0.91 ± 0.3) and a TSS of (0.71 ± 0.06), indicating strong predictive performance of all the 60 models (Table 2). The relative importance scores among the environmental variables ranged between 4.4 and 15.73%. Although the habitat suitability models exhibited high predictive performance, such metrics should be interpreted with caution. Very high values of AUC and related performance indices do not necessarily imply perfect ecological realism or predictive certainty, particularly for presence-only models applied to spatially structured, linear systems such as rivers.

**Table 2:** Performance Metrics of Different Modelling Methods. Comparison of various species distribution modelling methods, including Generalized Linear Models (GLM), Generalized Additive Models (GAM), Boosted Regression Trees (BRT), Random Forest (RF), Multivariate Adaptive Regression Splines (MARS), and Maximum Entropy (MaxEnt). The table presents key evaluation metrics: Area Under the Curve (AUC), Correlation Coefficient (COR), True Skill Statistic (TSS), and Deviance, which assess the predictive performance of each method.

Methods	AUC	COR	TSS	Deviance
GLM	0.88	0.59	0.63	0.68
GAM	0.9	0.67	0.71	1.43
BRT	0.9	0.65	0.66	0.71
RF	0.95	0.78	0.78	0.45
MARS	0.9	0.66	0.68	0.68
MaxEnt	0.92	0.68	0.71	0.61

**Fig 2:**
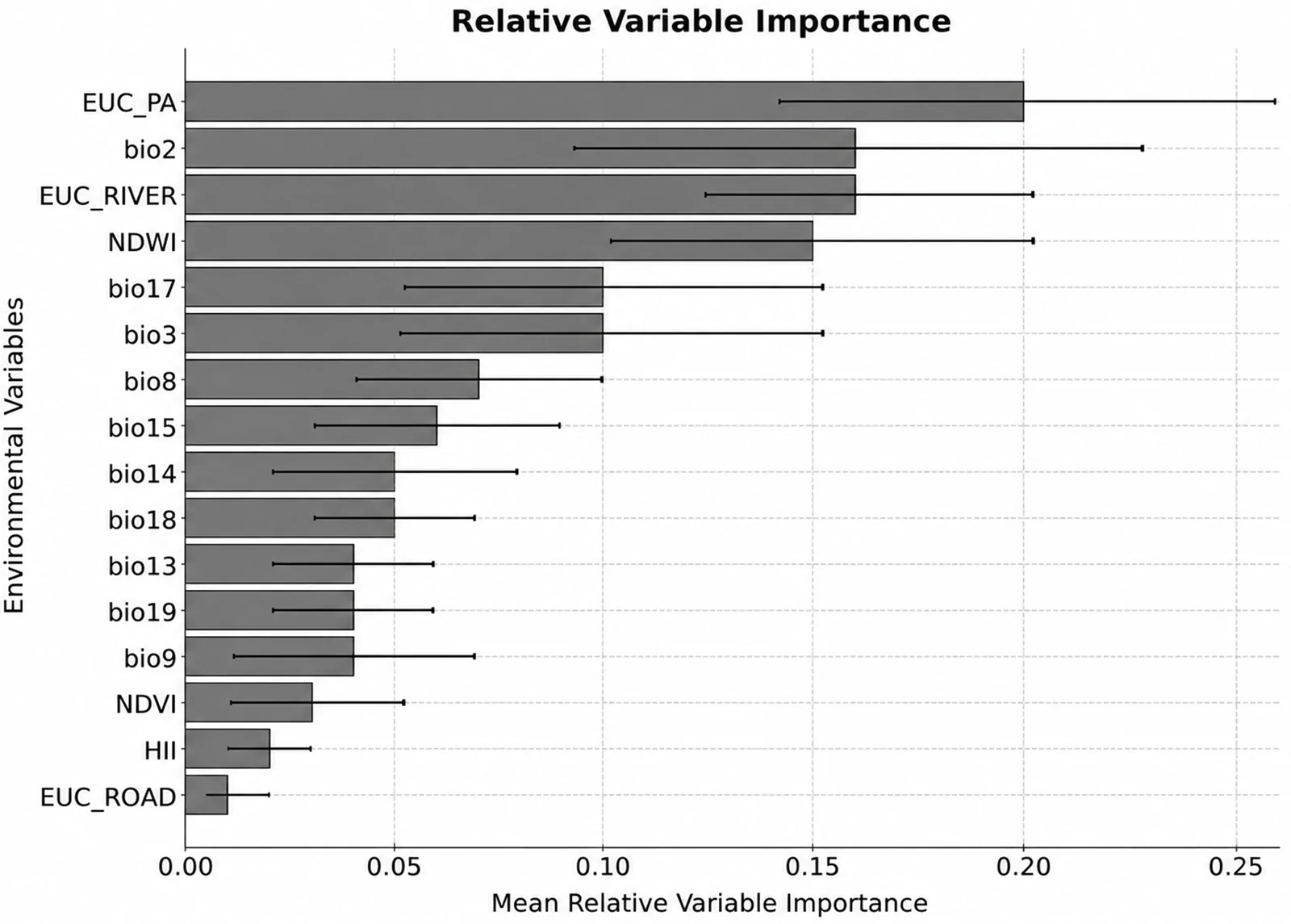
Relative Importance of Environmental Variables in Species Distribution Modelling. Bar plot illustrating the relative importance of environmental variables in the SDM.

The relative contribution of environmental variables to the species distribution model revealed that Euclidean distance to Protected Areas (EUC_PA) was the most influential predictor, with a mean relative importance of approximately 0.20, followed by Mean Diurnal Range (BIO2) at ~0.16 and Euclidean distance to river (EUC_RIVER) at ~0.15 (Fig 2). Other key variables included the Normalized Difference Water Index (NDWI) at ~0.14, and precipitation of driest quarter (BIO17) and Isothermality (BIO3), each contributing ~0.10. Together, these top six variables accounted for the dominant share of model prediction, emphasizing their collective influence in determining the distribution of Smooth-coated otter across the Ganga River Basin (Fig 2).

**Fig 3:**
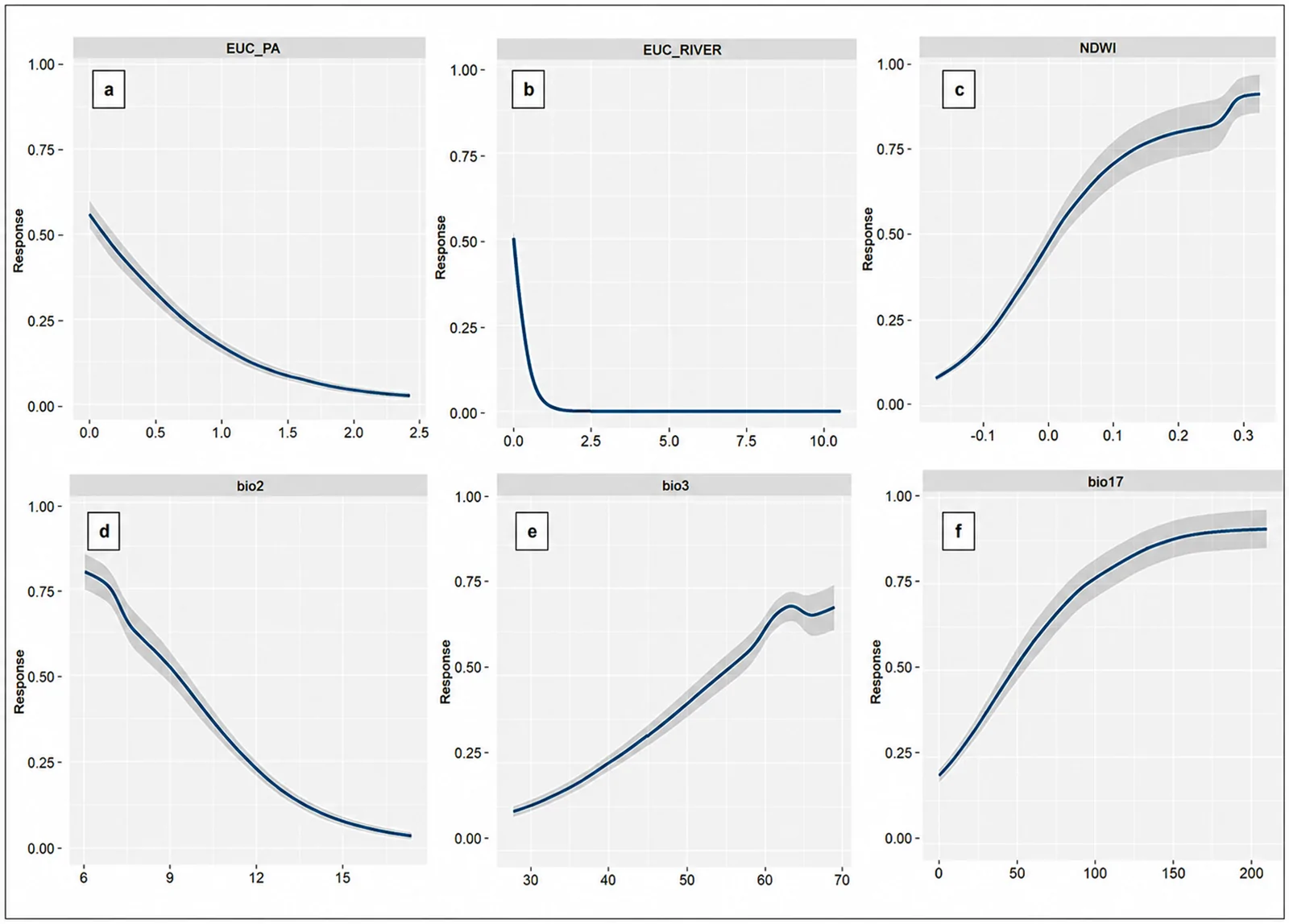
Relative Contribution of Key Environmental Variables to the Species Distribution Model. The most influential predictors include Euclidean distance to Protected Areas (EUC_PA), Euclidean distance to river (EUC_RIVER), Normalized Difference Water Index (NDWI), Mean Diurnal Range (BIO2), and Isothermality (BIO3). Response curves for each predictor variable illustrate their relationships with the predicted probability of species presence.

The response curve suggests a negative relationship between habitat suitability and distance from protected areas (Fig 3). Species occurrence probability is highest in areas closest to protected areas, with a gradual decline in suitability as the distance increases, with low suitability evident beyond 1 km. This indicates the species likely benefits from reduced anthropogenic disturbance or better habitat quality within or near protected areas like wildlife sanctuaries and tiger reserves.

Habitat suitability was also shown to be positive near river systems, with a sharp decline as the distance from rivers increased. Suitability decreased significantly beyond 2.5 km, indicating a strong dependence on proximity to aquatic ecosystems (Fig 3). Although occurrence records were collected exclusively along river systems, the Euclidean distance to river retained strong discriminatory power in the model because this variable captures the lateral riparian gradient distinguishing areas of immediate channel proximity used for foraging, resting, and denning from floodplain margins and upland hinterlands consistent with the known habitat use patterns of the species [35,40].The NDWI response showed a positive relationship, where the probability of species presence increased with higher NDWI values. This indicates that the species prefers areas with higher surface water content or moisture, consistent with aquatic or semi-aquatic habitat preferences. Suitability peaked at NDWI values above 0.25 (Fig 3).

Beyond confirming the species’ known aquatic association, these findings reveal ecologically meaningful thresholds and management-relevant nuances. The sharp decline in habitat suitability beyond 2.5 km from the nearest waterbody provides a quantitative riparian buffer threshold that can directly inform land-use zoning and the delineation of disturbance-free zones within identified otter conservation hotspots. Similarly, the NDWI threshold of >0.25 indicates that only river stretches and riparian zones with sustained surface moisture constitute functional habitat; seasonally dry, heavily abstracted, or hydrologically degraded channels fall below this threshold and are therefore unlikely to support stable otter populations regardless of their geographic proximity to rivers. Furthermore, the stronger relative importance of EUC_PA compared to EUC_RIVER suggests that mere proximity to water is insufficient for habitat suitability; protection status, reduced fishing pressure, and intact riparian structure collectively mediate occupancy beyond simple aquatic association. Together, these thresholds and hierarchical predictor relationships translate the species’ known ecological requirements into spatially explicit, quantitative criteria for conservation prioritization and habitat management across the GRB. The mean diurnal range (BIO2) had a negative association with the distribution of the species. As the range in temperature in a day increased, the suitability declined. The highest suitability was at the lowest range (6–8) and followed by a gradual decline thereafter, suggesting the species favours stable thermal environments with low daily fluctuations in temperature (Fig 3). This represents the species’ sensitivity towards temperature variations, which could be attributed to stress caused by the need to perform thermoregulation in fluctuating temperatures. The response curve for Isothermality (BIO3) revealed a positive correlation with habitat suitability. Suitability increased gradually with isothermality, suggesting that the species prefers areas where day-to-night temperature variation is moderate to high, relative to annual variation. The suitability had a positive relationship with precipitation during the driest quarter (BIO17) (Fig 3). Suitability increased with higher BIO17 (precipitation of the driest quarter) values, peaking at approximately 180–200 mm, indicating a preference for landscapes that retain water availability during seasonal dry phases. Sustained dry-season precipitation supports the persistence of shallow wetlands, perennial pools, and riparian backwaters, as critical microhabitats for prey. This dependence on aquatic prey bases, particularly in the dry season when river discharge contracts and alternative foraging refuges become essential for maintaining local prey availability and, consequently, habitat use.

**Fig 4:**
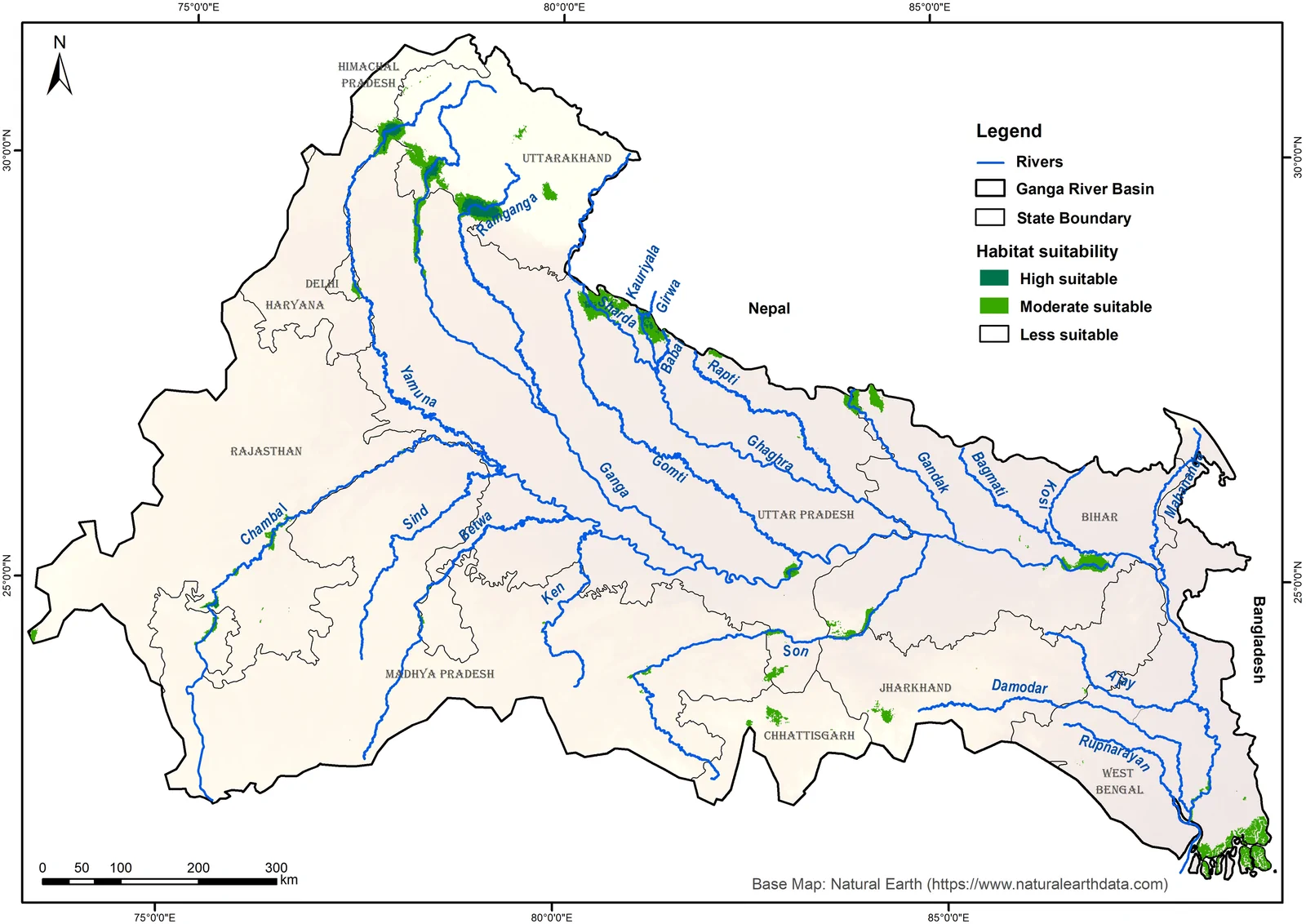
Predicted suitable habitats of Smooth-coated otter in the Ganga River Basin. Base Map: Natural Earth (www.naturalearthdata.com).

At the country level, the model predicted that approximately 35,27,982 km^2^ of the surveyed region is least suitable for Smooth-coated otter, while 115,739 km^2^ was categorized as moderately suitable and 32,444 km^2^ as highly suitable habitat (Fig 4). Within the Ganga River Basin, the model estimated that 806,698 km^2^ as least suitable, 13,300 km^2^ as moderately suitable, and 2,138 km^2^as highly suitable for Smooth-coated otter (Fig 4 and Table 3). High and moderate suitability habitats are predominantly concentrated in and around protected areas and their associated buffer zones, eco-sensitive zones, and notified conservation reserves in the Terai region, which collectively provide relatively undisturbed riparian zones with reduced anthropogenic pressure compared to unprotected river stretches (Fig 4).

**Table 3:** State-wise classification of habitat suitability (in km²) for Smooth-coated otter across selected states in northern and central India based on the Ensemble model. The area under each suitability category, least suitable, moderately suitable, and highly suitable, is provided for each state.

Name of Ganga Basin States	Least suitable (km²)	Moderate suitable (km²)	Highly suitable (km²)
Uttarakhand	48284.06	2417.11	1260.18
Uttar Pradesh	227213.25	4277.38	495.07
Himachal Pradesh	52781.18	1419.91	178.26
Haryana	42275.25	365.34	145.60
Rajasthan	329105.38	773.05	104.13
Bihar	88380.46	1954.69	79.42
Madhya Pradesh	295537.57	824.23	30.00
Jharkhand	75871.29	945.13	15.88
West Bengal	77157.95	2420.64	0
Chhattisgarh	129881.78	534.78	0
Delhi	1314.01	140.31	0

Since the Ganga River Basin spans multiple states, habitat suitability was also assessed at the state level. Among the states, Uttarakhand had the highest proportion of high-suitability habitat for Smooth-coated otter, with approximately 2.43% (1,260.18 km^2^) of its assessed area falling into this category. Uttar Pradesh emerged as the second most significant state, contributing 495.07 km^2^, which accounts for 0.21% of its total area. Himachal Pradesh followed with 0.33% (178.26 km^2^) of its area classified as highly suitable. Haryana supported 145.60 km^2^ of high suitability habitat, representing 0.34% of its surveyed area. Bihar contributed 79.42 km^2^, amounting to 0.088%, while Rajasthan and Madhya Pradesh had comparatively small proportions of highly suitable habitat, 0.032% (104.13 km^2^) and 0.010% (30.00 km^2^) respectively. Jharkhand had the least among states with recorded high suitability, contributing merely 15.88 km^2^ (0.021%), while West Bengal, Chhattisgarh, and NCT of Delhi recorded no highly suitable habitat (Table 3).

Notably, West Bengal, Chhattisgarh, and Delhi did not have any area classified as highly suitable for Smooth-coated otters (Fig 4 and Table 3). These variations reflect both ecological conditions and anthropogenic pressures across states and highlight the conservation importance of states like Uttarakhand, Uttar Pradesh, and Himachal Pradesh in maintaining viable otter populations within the Ganga River Basin. A portion of moderately suitable habitat of the Smooth-coated otter also falls under the National Capital Territory of Delhi (140.31 km^2^) (Fig 4 and Table 3), despite the region having a high human population density and pollution. The reason this region was selected to be moderately suitable by the model could be attributed to the presence of similar environmental conditions to some of the other habitats with high suitability. In some exceptional cases, as seen by their presence in a megacity such as Singapore, the species shows a high tolerance to human presence and disturbed landscapes. This could be the case only under specific conditions [36,89], as seen in the case of Singapore, where not only the adaptability of Smooth-coated otter accounts for their success, but also the strict upgraded laws, habitat restoration initiatives, and involvement of the public [90]. However, in contrast, there are only a few pockets in the National Capital Territory of Delhi, which could explain the lack of actual presence of Smooth-coated otter in the region. Thus, despite a model selecting an area as suitable, it does not assure the presence of the species in the area.

Further, the suitability was high in Rana Pratap Sagar, Gandhi Sagar, and the Jawahar Sagar dams on the Chambal River; Dakpathar, Hathnikund, Wazirabad, ITO, and Okhla barrages on the Yamuna; Indrapuri barrage on the Son; Harewali barrage and Ramganga and Kalagarh dams on the Ramganga; Tanakpur dam and Banbasa barrage on the Sharda, Kailashpuri dam on the Ghaghra; Gandak barrage on the Gandak; Panchet dam and Durgapur barrage on the Damodar; and Tehri dam, Bijnor, and Farakka barrage on the main stem Ganga. The suitability was also high in the Rajghat, Matatila, and Betwa river dams on the Betwa River, and Mohini Sagar (Madikhera) dam on the Sindh River, which are tributaries of the Yamuna River. Previous studies also noted the presence of otters in reservoirs created by dams and barrages, including Indonesia [91], Portugal [92], Brazil [93], and South Korea [94]. This could be attributed to these features having stable water presence, abundance in prey, and reduced human disturbances [95], which highlights the possibility that reservoirs help of the conservation of otters [93].

## Discussion

In the present study, we developed an ensemble SDM to predict the habitat suitability of Smooth-coated otter across the Ganga River Basin. Our results indicate that the distance to protected areas (EUC_PA), distance to river (EUC_River), Mean Diurnal Range (BIO2), Normalized Difference Water Index (NDWI), and Iso-thermality (BIO3) are the crucial contributors to the habitat suitability. Collectively, these predictors indicate that highly suitable habitats are characterized by proximity to PAs and perennial water sources, lower thermal variability, and stable hydrological conditions.

The strong association between high habitat suitability and proximity to protected areas likely reflects reduced anthropogenic disturbance, and better-preserved riparian vegetation, within or adjacent to protected landscapes [41]. Protected areas often function as refugia for otters by maintaining intact riverbanks, wetlands, and floodplain habitats, which are critical for resting, breeding, and foraging [100]. Moreover, the importance of low diurnal temperature fluctuations and moderate to high isothermality further suggests that Smooth-coated otter preferentially occupy thermally stable environments, which are likely to support consistent prey resources and reduce physiological stress, particularly during extreme seasonal conditions [112].

Our results indicate the potential distribution of Smooth-coated otter across foothill and piedmont river stretches (approximately 300–1,000 m asl) and lowland floodplains (below 300 m asl) of the basin, reflecting the species’ preference for transitional riverine zones that combine water permanence, moderate riparian structure, and comparatively lower anthropogenic pressure than densely populated plains. This suitability is likely due to these river stretches serving as ecological conduits for Smooth-coated otter, connecting fragmented habitats and facilitating movement between breeding, foraging, and refuge areas [35,96]. The resulting habitat suitability exhibits a fragmented distribution of suitable habitat across the study area, potentially due to natural riverine structure and varying degrees of anthropogenic modification [41,100].

The Terai region of India and Nepal consistently emerged as a zone of high habitat suitability, corroborating earlier studies that identified this region as an important stronghold for otter populations [97]. The left bank tributaries of the Ganga in Terai region exhibit the highest suitable habitats, particularly in the vicinity of protected areas such as Rajaji National Park, Dudhwa Tiger Reserve, Nadhaur Tiger Reserve, Corbett Tiger Reserve, Katerniaghat Wildlife Sanctuary along Girwa and Ghaghra, and Valmiki Tiger Reserve along Gandak. Similarly, the smaller rivers to the left of the Chambal and Son rivers are significant, indicating areas that range from fairly to very suitable along their middle and lower parts, explained by the presence of the National Chambal Sanctuary (NCS) and the Son Gharial Sanctuary [37,98]. These patterns likely reflect a combination of reduced human disturbance, intact riparian vegetation, availability of adjacent wetlands, and relatively continuous riverbanks features known to be critical for Smooth-coated otter [38,44,52,54]. In contrast, the absence of highly suitable habitats in several river stretches suggests that otters may increasingly rely on moderately suitable areas due to habitat loss and fragmentation. Anthropogenic pressures such as unregulated fishing, sand mining, riverbank agriculture, and infrastructure development can degrade habitat quality and reduce connectivity, forcing otters to occupy suboptimal habitats [41,52,99]. Such reliance on marginal habitats may increase vulnerability to human–wildlife conflict and elevate the risk of local population declines. Targeted habitat restoration in degraded stretches, regulating extractive activities, and maintaining flow regimes could therefore substantially enhance habitat suitability and connectivity for otters [40,100].

Notably, about 21% of the suitable habitat occur outside the boundaries of existing PAs, underscoring the limitations of protected-area-centric conservation strategies and bears a significant influence on the long-term conservation strategies (Fig 4). Buffer zones around the protected areas often experience encroachment, construction activities, insufficient ecological regulations, sand mining, agriculture, and unregulated fishing, which makes our study area highly susceptible to human and animal conflict and conservation of otter habitat in the basin. Hence, conservation strategies should not only include protected areas but also community-managed areas, riverbank protections, and land-use planning with the involvement of stakeholders [48,41]. The PAs, such as the National Chambal Sanctuary and the Vikramshila Gangetic Dolphin Sanctuary, showed a larger portion of highly suitable habitats (Fig 4). These areas benefit from low human interference and rich prey diversity, largely resulting from fishing restrictions and low human disturbances which together contribute to the maintenance of stable otter populations, highlighting the importance of protected areas in the otter conservation. Additionally, several other rivers, including Ghaghra, Sharda, Ramganga, and Gandak, support suitable habitats outside protected areas while simultaneously facing intense anthropogenic pressures. Illegal sand mining and riverbed agriculture along the Chambal and the mainstem Ganga cause riverbank erosion and degradation of fish spawning and refuge habitats [101]. Pollution from industrial and agricultural effluents in the mainstem Ganga further reduces prey availability, particularly fish and amphibians [28,102]. Additionally, dams and barrages disrupt natural flow regimes and seasonal flooding, which are essential for wetland regeneration and prey productivity, while also restricting otter movement and dispersal [103,104]. Conflicts between otters and fishing communities are widespread across tributaries, and the replacement of native riparian vegetation with monoculture plantations or agriculture has further reduced resting and breeding habitats [48,105]. Collectively, these pressures reduce habitat quality and connectivity, increasing the likelihood of population fragmentation and local extinctions [106,107].

Studies conducted along the Chambal and Ganga rivers [48], as well as in the small rivers in the Western Himalayan foothills [98], has consistently demonstrated that otters prefers open canopy riparian zones, dense wet grasslands, and proximity to both seasonal and perennial wetlands. Our research also supports these findings, showing that the highly suitable areas for otters are those with moderate to low NDVI values, which aligns with the fact that this species tends to stay away from dense canopy [98]. The link between rainfall during dry times and the quality of habitats matches the findings in Nepal [41], which suggest that changes in water levels are important for the survival of otters. In the Ganga River basin and adjacent areas, there is a significant lack of systematic research on otter population dynamics [41,108]. Reports from fishing communities in Sahibganj in the state of Jharkhand and quick checks by conservation groups support the idea that there are small, scattered otter populations. Due to significant fragmentation and human impact in the area, immediate conservation measures are necessary to safeguard and link appropriate otter habitats [48,109].

This study provides a basin-scale assessment of habitat suitability for Smooth-coated otters; however, several limitations should be considered. Habitat suitability was modelled using presence-only data, and detection probability was not explicitly accounted for, potentially leading to an underestimation of otter occurrence in some river stretches [110, 111]. Additionally, the predictor variables represent broad-scale environmental and anthropogenic conditions and may not capture fine-scale habitat features or temporal dynamics associated with seasonal flows and water regulation [112, 113].

Future studies should incorporate repeated surveys to account for detection probability, finer-scale habitat assessments, and movement or genetic data to improve understanding of connectivity and population structure. Integrating temporal hydrological variability and socio-ecological factors will further strengthen conservation planning and the translation of habitat suitability outputs into effective management actions [114, 115]. A further limitation of this study is the absence of direct prey availability data. Instead, variables such as NDWI, BIO17 (precipitation of the driest quarter), proximity to protected areas, and human influence index were used as indirect proxies for aquatic productivity and food resource availability. While this approach is consistent with basin-scale SDM studies where direct prey data are rarely available, future studies should incorporate prey abundance or biomass data as explicit predictors to improve the ecological realism of habitat suitability models.

### Conservation implications and conclusion

Effective conservation planning for the Smooth-coated otter in the Ganga River Basin (GRB) must integrate spatially explicit information on habitat suitability with population vulnerability, anthropogenic pressures, and the feasibility of long-term management interventions [93]. The results of this study identify discrete yet fragmented riverine habitats that are critical for otters and emphasize the need for targeted, landscape-scale conservation actions rather than isolated, site-based efforts. Highly suitable habitats in the Terai region should be prioritized for conservation through the designation of community reserves and other community-managed conservation frameworks. Such approaches can be particularly effective in these riverine landscapes where a substantial proportion of suitable habitats lie outside PAs. Furthermore, community-based conservation initiatives, combined conflict-mitigation strategies, are essential for reducing human–otter conflicts, particularly in fishing-dominated landscapes [10,24].

The fragmented distribution of suitable habitats across the basin highlights the urgency of protecting remaining otter habitats and maintaining longitudinal and lateral river connectivity to support dispersal, gene flow, and long-term population persistence. Immediate management actions are required to regulate or prohibit sand mining, riverbed agriculture, and encroachments along riverbanks within identified otter hotspots [105]. In areas where complete bans are not socioeconomically feasible, regulated sand extraction and rotational harvesting under strict monitoring frameworks should be implemented to minimize ecological impacts [105]. Additionally, human–otter conflicts involving fishing communities should be addressed through compensation mechanisms, education and awareness programmes, and the promotion of alternative livelihood options to reduce dependency on riverine resources.

Overall, the long-term persistence of Smooth-coated otter populations in the GRB will require coordinated conservation actions that extend beyond protected areas, enforce habitat protection measures, and actively promote community-based stewardship of riverine ecosystems. Future conservation initiatives should emphasize policy integration, cross-sectoral coordination, and stakeholder-driven implementation to translate habitat suitability assessments into measurable conservation outcomes [116,117]. Embedding otter-specific management strategies within existing wetland, river, and fisheries management programmes and aligning land-use, sand mining, and fisheries policies with biodiversity objectives will be critical for reducing habitat degradation and mitigating human–otter conflicts across the basin [10,118,119].

## Supporting information

S1 File Supporting figures and tables for SDM. Includes ROC curves comparing the predictive performance of six modelling algorithms (GLM, GAM, BRT, RF, MARS, and MaxEnt) under two resampling methods; a calibration plot, threshold selection plot (based on the TSS), density plot, and boxplot of predicted probabilities for the RF model; and three tables summarizing model performance metrics, model replication and validation details, and presence locations used following spatial thinning of occurrence records.
(DOCX)
