# Supplementary material for: Beyond sightings: Ensemble modelling to identify conservation hotspots of Smooth-coated otters (Lutrogale perspicillata) in the Ganga River Basin for informed policy and sustainability
Source: PLoS One. 2026 Aug 19;21(8):e0353661. doi: 10.1371/journal.pone.0353661 (PMC13489423; doi:10.1371/journal.pone.0353661)
Supplement: Supplementary file 1 [file pone.0353661.s001.docx]

**(PONE-D-25-50748)**

**“Beyond Sightings: Ensemble Modelling to Identify Conservation Hotspots of Smooth-Coated Otters (Lutrogale perspicillata) in the Ganga River Basin for Informed Policy and Sustainability.”**

**Supplementary material**

**S1 File**. Supporting figures and tables for SDM. Includes ROC curves comparing the predictive performance of six modelling algorithms (GLM, GAM, BRT, RF, MARS, and MaxEnt) under two resampling methods; a calibration plot, threshold selection plot (based on the TSS), density plot, and boxplot of predicted probabilities for the RF model; and three tables summarizing model performance metrics, model replication and validation details, and presence locations used following spatial thinning of occurrence records.

| **Model ID** | **AUC** | **COR** | **Deviance** | **TSS** | **MCC** | **F1** |
| --- | --- | --- | --- | --- | --- | --- |
| 38 | 0.98 | 0.869 | 0.351721 | 0.8788 | 0.8814 | 0.9099 |
| 36 | 0.976 | 0.864 | 0.354821 | 0.8595 | 0.8371 | 0.8755 |
| 37 | 0.97 | 0.843 | 0.397187 | 0.8273 | 0.7922 | 0.8446 |
| 40 | 0.968 | 0.838 | 0.407603 | 0.8241 | 0.7941 | 0.8455 |
| 39 | 0.959 | 0.828 | 0.41114 | 0.8474 | 0.8008 | 0.8477 |
| 32 | 0.944 | 0.762 | 0.53344 | 0.7446 | 0.6788 | 0.7586 |
| 26 | 0.941 | 0.731 | 0.736596 | 0.7471 | 0.663 | 0.7384 |
| 56 | 0.939 | 0.742 | 0.584636 | 0.7697 | 0.702 | 0.771 |
| 35 | 0.938 | 0.76 | 0.535432 | 0.7667 | 0.7542 | 0.8159 |
| 34 | 0.936 | 0.773 | 0.535911 | 0.7652 | 0.6904 | 0.7647 |
| 33 | 0.934 | 0.749 | 0.560931 | 0.7611 | 0.7064 | 0.7803 |
| 58 | 0.934 | 0.719 | 0.617632 | 0.7414 | 0.6862 | 0.764 |
| 57 | 0.93 | 0.722 | 0.615011 | 0.7515 | 0.6843 | 0.7626 |
| 60 | 0.926 | 0.716 | 0.629525 | 0.7065 | 0.7005 | 0.7745 |
| 19 | 0.923 | 0.77 | 1.108022 | 0.7608 | 0.7681 | 0.8219 |
| 31 | 0.921 | 0.733 | 0.58919 | 0.7348 | 0.6619 | 0.7448 |
| 28 | 0.919 | 0.707 | 0.7565 | 0.678 | 0.6458 | 0.7352 |
| 52 | 0.918 | 0.696 | 0.6721 | 0.7376 | 0.6803 | 0.7611 |
| 47 | 0.917 | 0.699 | 0.741677 | 0.7227 | 0.6737 | 0.7576 |
| 48 | 0.915 | 0.675 | 0.630076 | 0.6882 | 0.6081 | 0.7023 |
| 20 | 0.913 | 0.747 | 1.183814 | 0.7207 | 0.7165 | 0.7863 |
| 24 | 0.913 | 0.722 | 0.793036 | 0.7061 | 0.6822 | 0.7633 |
| 27 | 0.913 | 0.68 | 0.7913 | 0.6837 | 0.6125 | 0.7103 |
| 54 | 0.913 | 0.695 | 0.685658 | 0.7338 | 0.6873 | 0.7671 |
| 17 | 0.912 | 0.723 | 0.757479 | 0.7285 | 0.672 | 0.7556 |
| 46 | 0.912 | 0.691 | 0.61151 | 0.6882 | 0.6142 | 0.7055 |
| 50 | 0.912 | 0.682 | 0.757908 | 0.6916 | 0.6359 | 0.7286 |
| 12 | 0.911 | 0.703 | 0.708949 | 0.7099 | 0.673 | 0.757 |
| 30 | 0.911 | 0.662 | 0.801335 | 0.6813 | 0.6114 | 0.7088 |
| 59 | 0.911 | 0.691 | 0.638943 | 0.6982 | 0.6296 | 0.7191 |
| 51 | 0.91 | 0.691 | 0.672117 | 0.7219 | 0.6393 | 0.7251 |
| 6 | 0.908 | 0.651 | 0.680203 | 0.7294 | 0.6629 | 0.7424 |
| 42 | 0.908 | 0.686 | 0.660121 | 0.6882 | 0.608 | 0.7036 |
| 53 | 0.906 | 0.672 | 0.706667 | 0.7219 | 0.6393 | 0.7251 |
| 45 | 0.905 | 0.696 | 0.638068 | 0.694 | 0.6293 | 0.7234 |
| 15 | 0.904 | 0.692 | 0.771573 | 0.7169 | 0.6699 | 0.7545 |
| 8 | 0.902 | 0.634 | 0.691431 | 0.6832 | 0.5879 | 0.6766 |
| 29 | 0.902 | 0.64 | 0.790486 | 0.6946 | 0.6025 | 0.6913 |
| 55 | 0.9 | 0.67 | 0.690153 | 0.714 | 0.6546 | 0.7424 |
| 44 | 0.898 | 0.685 | 0.691469 | 0.6618 | 0.653 | 0.74 |
| 14 | 0.897 | 0.704 | 0.881061 | 0.6924 | 0.6768 | 0.7586 |
| 10 | 0.896 | 0.626 | 0.717897 | 0.6776 | 0.59 | 0.6857 |
| 22 | 0.896 | 0.659 | 0.809344 | 0.6763 | 0.632 | 0.7273 |
| 18 | 0.894 | 0.715 | 1.783 | 0.7433 | 0.7003 | 0.7752 |
| 41 | 0.894 | 0.661 | 0.70416 | 0.6997 | 0.665 | 0.7512 |
| 49 | 0.893 | 0.671 | 0.737576 | 0.6893 | 0.6427 | 0.7309 |
| 7 | 0.891 | 0.618 | 0.712462 | 0.6417 | 0.5638 | 0.6731 |
| 16 | 0.89 | 0.742 | 4.786644 | 0.7665 | 0.7466 | 0.8069 |
| 25 | 0.889 | 0.633 | 0.8256 | 0.6776 | 0.6057 | 0.7049 |
| 1 | 0.887 | 0.634 | 0.718991 | 0.6434 | 0.5805 | 0.6891 |
| 11 | 0.887 | 0.688 | 0.746338 | 0.6786 | 0.6719 | 0.7538 |
| 21 | 0.885 | 0.642 | 0.821384 | 0.6528 | 0.6059 | 0.7085 |
| 13 | 0.884 | 0.666 | 1.189009 | 0.6522 | 0.6231 | 0.7204 |
| 23 | 0.884 | 0.638 | 0.829226 | 0.6544 | 0.5769 | 0.6824 |
| 2 | 0.88 | 0.602 | 0.760134 | 0.6715 | 0.59 | 0.6899 |
| 4 | 0.873 | 0.62 | 0.74496 | 0.6045 | 0.5874 | 0.6927 |
| 5 | 0.873 | 0.602 | 0.761814 | 0.654 | 0.5822 | 0.6883 |
| 3 | 0.87 | 0.576 | 0.804013 | 0.6517 | 0.5676 | 0.6716 |
| 43 | 0.869 | 0.626 | 0.78624 | 0.6305 | 0.5591 | 0.672 |
| 9 | 0.865 | 0.582 | 0.808743 | 0.6496 | 0.5779 | 0.6812 |

| modelID | species | method | replication | ID | success | training | test.dep | test.indep |
| --- | --- | --- | --- | --- | --- | --- | --- | --- |
| 1 | sp | glm | subsampling | 1 | True | True | True | False |
| 2 | sp | glm | subsampling | 2 | True | True | True | False |
| 3 | sp | glm | subsampling | 3 | True | True | True | False |
| 4 | sp | glm | subsampling | 4 | True | True | True | False |
| 5 | sp | glm | subsampling | 5 | True | True | True | False |
| 6 | sp | glm | bootstrap | 6 | True | True | True | False |
| 7 | sp | glm | bootstrap | 7 | True | True | True | False |
| 8 | sp | glm | bootstrap | 8 | True | True | True | False |
| 9 | sp | glm | bootstrap | 9 | True | True | True | False |
| 10 | sp | glm | bootstrap | 10 | True | True | True | False |
| 11 | sp | gam | subsampling | 1 | True | True | True | False |
| 12 | sp | gam | subsampling | 2 | True | True | True | False |
| 13 | sp | gam | subsampling | 3 | True | True | True | False |
| 14 | sp | gam | subsampling | 4 | True | True | True | False |
| 15 | sp | gam | subsampling | 5 | True | True | True | False |
| 16 | sp | gam | bootstrap | 6 | True | True | True | False |
| 17 | sp | gam | bootstrap | 7 | True | True | True | False |
| 18 | sp | gam | bootstrap | 8 | True | True | True | False |
| 19 | sp | gam | bootstrap | 9 | True | True | True | False |
| 20 | sp | gam | bootstrap | 10 | True | True | True | False |
| 21 | sp | brt | subsampling | 1 | True | True | True | False |
| 22 | sp | brt | subsampling | 2 | True | True | True | False |
| 23 | sp | brt | subsampling | 3 | True | True | True | False |
| 24 | sp | brt | subsampling | 4 | True | True | True | False |
| 25 | sp | brt | subsampling | 5 | True | True | True | False |
| 26 | sp | brt | bootstrap | 6 | True | True | True | False |
| 27 | sp | brt | bootstrap | 7 | True | True | True | False |
| 28 | sp | brt | bootstrap | 8 | True | True | True | False |
| 29 | sp | brt | bootstrap | 9 | True | True | True | False |
| 30 | sp | brt | bootstrap | 10 | True | True | True | False |
| 31 | sp | rf | subsampling | 1 | True | True | True | False |
| 32 | sp | rf | subsampling | 2 | True | True | True | False |
| 33 | sp | rf | subsampling | 3 | True | True | True | False |
| 34 | sp | rf | subsampling | 4 | True | True | True | False |
| 35 | sp | rf | subsampling | 5 | True | True | True | False |
| 36 | sp | rf | bootstrap | 6 | True | True | True | False |
| 37 | sp | rf | bootstrap | 7 | True | True | True | False |
| 38 | sp | rf | bootstrap | 8 | True | True | True | False |
| 39 | sp | rf | bootstrap | 9 | True | True | True | False |
| 40 | sp | rf | bootstrap | 10 | True | True | True | False |
| 41 | sp | mars | subsampling | 1 | True | True | True | False |
| 42 | sp | mars | subsampling | 2 | True | True | True | False |
| 43 | sp | mars | subsampling | 3 | True | True | True | False |
| 44 | sp | mars | subsampling | 4 | True | True | True | False |
| 45 | sp | mars | subsampling | 5 | True | True | True | False |
| 46 | sp | mars | bootstrap | 6 | True | True | True | False |
| 47 | sp | mars | bootstrap | 7 | True | True | True | False |
| 48 | sp | mars | bootstrap | 8 | True | True | True | False |
| 49 | sp | mars | bootstrap | 9 | True | True | True | False |
| 50 | sp | mars | bootstrap | 10 | True | True | True | False |
| 51 | sp | maxent | subsampling | 1 | True | True | True | False |
| 52 | sp | maxent | subsampling | 2 | True | True | True | False |
| 53 | sp | maxent | subsampling | 3 | True | True | True | False |
| 54 | sp | maxent | subsampling | 4 | True | True | True | False |
| 55 | sp | maxent | subsampling | 5 | True | True | True | False |
| 56 | sp | maxent | bootstrap | 6 | True | True | True | False |
| 57 | sp | maxent | bootstrap | 7 | True | True | True | False |
| 58 | sp | maxent | bootstrap | 8 | True | True | True | False |
| 59 | sp | maxent | bootstrap | 9 | True | True | True | False |
| 60 | sp | maxent | bootstrap | 10 | True | True | True | False |

| sp | long | lat |
| --- | --- | --- |
| *Lutrogale perspicillata* | 79.09518 | 29.55616 |
| *Lutrogale perspicillata* | 77.72978 | 11.93158 |
| *Lutrogale perspicillata* | 78.82339 | 10.83771 |
| *Lutrogale perspicillata* | 92.82859 | 26.59219 |
| *Lutrogale perspicillata* | 78.91581 | 10.85848 |
| *Lutrogale perspicillata* | 76.2725 | 11.83726 |
| *Lutrogale perspicillata* | 75.76241 | 13.67921 |
| *Lutrogale perspicillata* | 75.57203 | 13.71261 |
| *Lutrogale perspicillata* | 93.27112 | 26.62667 |
| *Lutrogale perspicillata* | 93.28509 | 26.67065 |
| *Lutrogale perspicillata* | 76.84225 | 10.29845 |
| *Lutrogale perspicillata* | 74.88377 | 31.14602 |
| *Lutrogale perspicillata* | 76.04748 | 25.32456 |
| *Lutrogale perspicillata* | 93.33379 | 26.74258 |
| *Lutrogale perspicillata* | 93.5946 | 26.75013 |
| *Lutrogale perspicillata* | 73.92373 | 15.53989 |
| *Lutrogale perspicillata* | 78.98886 | 29.47771 |
| *Lutrogale perspicillata* | 76.60719 | 11.59463 |
| *Lutrogale perspicillata* | 93.35178 | 26.72714 |
| *Lutrogale perspicillata* | 74.19347 | 14.94477 |
| *Lutrogale perspicillata* | 93.44853 | 26.68687 |
| *Lutrogale perspicillata* | 79.14239 | 29.46205 |
| *Lutrogale perspicillata* | 93.54292 | 26.7604 |
| *Lutrogale perspicillata* | 78.93419 | 29.51664 |
| *Lutrogale perspicillata* | 76.68082 | 8.845203 |
| *Lutrogale perspicillata* | 93.36781 | 26.77759 |
| *Lutrogale perspicillata* | 93.30891 | 26.77242 |
| *Lutrogale perspicillata* | 76.15611 | 11.90758 |
| *Lutrogale perspicillata* | 93.37895 | 26.45893 |
| *Lutrogale perspicillata* | 74.17291 | 15.01449 |
| *Lutrogale perspicillata* | 93.08754 | 26.53225 |
| *Lutrogale perspicillata* | 93.04888 | 26.49194 |
| *Lutrogale perspicillata* | 76.29151 | 15.20742 |
| *Lutrogale perspicillata* | 77.33277 | 12.29124 |
| *Lutrogale perspicillata* | 92.11439 | 27.52774 |
| *Lutrogale perspicillata* | 93.39022 | 26.75022 |
| *Lutrogale perspicillata* | 76.20861 | 11.92144 |
| *Lutrogale perspicillata* | 77.67015 | 11.99453 |
| *Lutrogale perspicillata* | 77.06316 | 9.595946 |
| *Lutrogale perspicillata* | 78.53975 | 26.9619 |
| *Lutrogale perspicillata* | 76.24384 | 11.95071 |
| *Lutrogale perspicillata* | 76.43146 | 15.27262 |
| *Lutrogale perspicillata* | 74.35753 | 15.27353 |
| *Lutrogale perspicillata* | 73.91835 | 15.44901 |
| *Lutrogale perspicillata* | 76.0786 | 12.13469 |
| *Lutrogale perspicillata* | 73.88377 | 15.51467 |
| *Lutrogale perspicillata* | 80.11148 | 28.6941 |
| *Lutrogale perspicillata* | 76.22385 | 11.86246 |
| *Lutrogale perspicillata* | 76.09577 | 12.08442 |
| *Lutrogale perspicillata* | 76.06532 | 12.1603 |
| *Lutrogale perspicillata* | 77.62085 | 12.70588 |
| *Lutrogale perspicillata* | 77.64823 | 12.68033 |
| *Lutrogale perspicillata* | 77.60197 | 12.66286 |
| *Lutrogale perspicillata* | 77.66068 | 11.87857 |
| *Lutrogale perspicillata* | 81.67006 | 17.19348 |
| *Lutrogale perspicillata* | 76.13681 | 11.75074 |
| *Lutrogale perspicillata* | 77.36546 | 8.713131 |
| *Lutrogale perspicillata* | 73.92863 | 15.40575 |
| *Lutrogale perspicillata* | 76.93108 | 10.30612 |
| *Lutrogale perspicillata* | 76.88589 | 10.36467 |
| *Lutrogale perspicillata* | 76.22648 | 11.95801 |
| *Lutrogale perspicillata* | 75.44874 | 24.9134 |
| *Lutrogale perspicillata* | 76.04343 | 12.16619 |
| *Lutrogale perspicillata* | 76.15621 | 12.01128 |
| *Lutrogale perspicillata* | 76.11346 | 12.01442 |
| *Lutrogale perspicillata* | 76.03633 | 12.0061 |
| *Lutrogale perspicillata* | 80.71642 | 16.51501 |
| *Lutrogale perspicillata* | 80.61366 | 16.49622 |
| *Lutrogale perspicillata* | 76.98898 | 10.22701 |
| *Lutrogale perspicillata* | 93.06552 | 26.57158 |
| *Lutrogale perspicillata* | 77.04123 | 9.408133 |
| *Lutrogale perspicillata* | 77.17979 | 9.572952 |
| *Lutrogale perspicillata* | 93.07182 | 26.41022 |
| *Lutrogale perspicillata* | 77.14778 | 9.42165 |
| *Lutrogale perspicillata* | 74.84415 | 13.81636 |
| *Lutrogale perspicillata* | 77.38919 | 9.418338 |
| *Lutrogale perspicillata* | 90.89616 | 26.6024 |
| *Lutrogale perspicillata* | 80.56935 | 16.50621 |
| *Lutrogale perspicillata* | 77.66548 | 12.06361 |
| *Lutrogale perspicillata* | 76.65635 | 12.42436 |
| *Lutrogale perspicillata* | 76.84217 | 12.32489 |
| *Lutrogale perspicillata* | 78.81884 | 29.49861 |
| *Lutrogale perspicillata* | 80.52454 | 28.25781 |
| *Lutrogale perspicillata* | 76.78326 | 12.39636 |
| *Lutrogale perspicillata* | 76.72059 | 12.4069 |
| *Lutrogale perspicillata* | 77.06685 | 9.477606 |
| *Lutrogale perspicillata* | 76.55744 | 9.428437 |
| *Lutrogale perspicillata* | 87.97726 | 24.92223 |
| *Lutrogale perspicillata* | 77.11236 | 9.455285 |
| *Lutrogale perspicillata* | 77.43217 | 12.28802 |
| *Lutrogale perspicillata* | 73.05136 | 22.36408 |
| *Lutrogale perspicillata* | 80.42153 | 28.37053 |
| *Lutrogale perspicillata* | 75.8291 | 11.1309 |
| *Lutrogale perspicillata* | 76.68646 | 12.12687 |
| *Lutrogale perspicillata* | 82.30361 | 16.8062 |
| *Lutrogale perspicillata* | 78.9503 | 29.52478 |
| *Lutrogale perspicillata* | 76.71636 | 12.08889 |
| *Lutrogale perspicillata* | 75.99888 | 10.86577 |
| *Lutrogale perspicillata* | 73.99734 | 15.48602 |
| *Lutrogale perspicillata* | 76.04363 | 10.89625 |
| *Lutrogale perspicillata* | 79.13476 | 29.35873 |
| *Lutrogale perspicillata* | 77.45926 | 12.27307 |
| *Lutrogale perspicillata* | 93.2563 | 26.60875 |
| *Lutrogale perspicillata* | 92.98017 | 26.40359 |
| *Lutrogale perspicillata* | 93.02484 | 26.58289 |
| *Lutrogale perspicillata* | 93.02059 | 26.54314 |
| *Lutrogale perspicillata* | 74.00114 | 15.33021 |
| *Lutrogale perspicillata* | 73.84046 | 15.50994 |
| *Lutrogale perspicillata* | 76.34154 | 11.20237 |
| *Lutrogale perspicillata* | 77.57515 | 12.2042 |
| *Lutrogale perspicillata* | 93.46667 | 26.6 |
| *Lutrogale perspicillata* | 77.61498 | 12.21897 |
| *Lutrogale perspicillata* | 77.68913 | 12.20017 |
| *Lutrogale perspicillata* | 93.10756 | 26.53324 |
| *Lutrogale perspicillata* | 93.20682 | 26.40687 |
| *Lutrogale perspicillata* | 93.55079 | 26.69258 |
| *Lutrogale perspicillata* | 93.27542 | 26.6414 |
| *Lutrogale perspicillata* | 92.88967 | 26.60569 |
| *Lutrogale perspicillata* | 73.93614 | 15.55941 |
| *Lutrogale perspicillata* | 93.21866 | 26.73034 |
| *Lutrogale perspicillata* | 73.31059 | 16.72733 |
| *Lutrogale perspicillata* | 93.19617 | 26.52204 |
| *Lutrogale perspicillata* | 76.43188 | 15.3595 |
| *Lutrogale perspicillata* | 76.19525 | 10.41085 |
| *Lutrogale perspicillata* | 93.34391 | 26.6903 |
| *Lutrogale perspicillata* | 93.31508 | 26.4235 |
| *Lutrogale perspicillata* | 93.34712 | 26.70418 |
| *Lutrogale perspicillata* | 82.2915 | 16.98678 |
| *Lutrogale perspicillata* | 76.31058 | 11.98488 |
| *Lutrogale perspicillata* | 76.20455 | 11.81557 |
| *Lutrogale perspicillata* | 77.00501 | 9.469006 |
| *Lutrogale perspicillata* | 93.14226 | 26.4189 |
| *Lutrogale perspicillata* | 93.29453 | 26.60879 |
| *Lutrogale perspicillata* | 95.22216 | 27.83656 |
| *Lutrogale perspicillata* | 78.14143 | 22.57522 |
| *Lutrogale perspicillata* | 90.97884 | 26.68142 |
| *Lutrogale perspicillata* | 74.55505 | 16.11051 |
| *Lutrogale perspicillata* | 72.84625 | 19.0123 |
| *Lutrogale perspicillata* | 78.65494 | 17.31414 |
| *Lutrogale perspicillata* | 82.04076 | 17.32101 |
| *Lutrogale perspicillata* | 73.1201 | 20.39668 |
| *Lutrogale perspicillata* | 77.56194 | 32.10241 |
| *Lutrogale perspicillata* | 72.90043 | 21.24581 |
| *Lutrogale perspicillata* | 80.83863 | 26.03453 |
| *Lutrogale perspicillata* | 80.80428 | 27.1787 |
| *Lutrogale perspicillata* | 79.21201 | 26.43991 |
| *Lutrogale perspicillata* | 82.547 | 26.649 |
| *Lutrogale perspicillata* | 84.528 | 25.828 |
| *Lutrogale perspicillata* | 80.0831 | 28.75064 |
| *Lutrogale perspicillata* | 78.52699 | 29.69398 |
| *Lutrogale perspicillata* | 78.8417 | 29.45927 |
| *Lutrogale perspicillata* | 79.19381 | 29.52635 |
| *Lutrogale perspicillata* | 79.3013 | 30.31598 |
| *Lutrogale perspicillata* | 79.28257 | 30.44868 |
| *Lutrogale perspicillata* | 78.64188 | 29.98919 |
| *Lutrogale perspicillata* | 80.56274 | 22.12209 |
| *Lutrogale perspicillata* | 80.58269 | 22.19018 |
| *Lutrogale perspicillata* | 80.06914 | 22.06401 |
| *Lutrogale perspicillata* | 88.82751 | 22.12599 |
| *Lutrogale perspicillata* | 88.7662 | 22.08774 |
| *Lutrogale perspicillata* | 81.09814 | 23.59124 |
| *Lutrogale perspicillata* | 78.62679 | 29.87447 |
| *Lutrogale perspicillata* | 78.93868 | 29.5548 |
| *Lutrogale perspicillata* | 78.21624 | 29.78884 |
| *Lutrogale perspicillata* | 78.09628 | 29.16522 |
| *Lutrogale perspicillata* | 78.64289 | 30.20095 |
| *Lutrogale perspicillata* | 86.73593 | 25.25704 |
| *Lutrogale perspicillata* | 82.09819 | 24.95392 |
| *Lutrogale perspicillata* | 77.86357 | 26.60742 |
| *Lutrogale perspicillata* | 75.61737 | 24.56458 |
| *Lutrogale perspicillata* | 75.62227 | 24.8348 |
| *Lutrogale perspicillata* | 82.14073 | 24.51194 |
| *Lutrogale perspicillata* | 76.85603 | 11.27749 |
| *Lutrogale perspicillata* | 75.97978 | 11.28426 |
| *Lutrogale perspicillata* | 75.87292 | 11.21057 |
| *Lutrogale perspicillata* | 92.81846 | 27.09547 |
| *Lutrogale perspicillata* | 92.63467 | 27.02733 |
| *Lutrogale perspicillata* | 92.16009 | 22.71268 |
| *Lutrogale perspicillata* | 76.45993 | 15.33769 |
| *Lutrogale perspicillata* | 73.86193 | 15.51914 |
| *Lutrogale perspicillata* | 78.34054 | 9.797286 |
| *Lutrogale perspicillata* | 92.73378 | 26.61975 |
| *Lutrogale perspicillata* | 78.76418 | 19.12366 |
| *Lutrogale perspicillata* | 79.01557 | 19.07521 |
| *Lutrogale perspicillata* | 78.99656 | 19.09313 |
| *Lutrogale perspicillata* | 79.01188 | 19.12697 |
| *Lutrogale perspicillata* | 78.96438 | 19.07916 |
| *Lutrogale perspicillata* | 74.714 | 13.55426 |
| *Lutrogale perspicillata* | 77.30914 | 12.78887 |
| *Lutrogale perspicillata* | 77.49 | 13.15028 |
| *Lutrogale perspicillata* | 73.2452 | 19.3005 |
| *Lutrogale perspicillata* | 73.08693 | 19.04254 |
| *Lutrogale perspicillata* | 73.38418 | 16.60055 |
| *Lutrogale perspicillata* | 73.3996 | 16.59119 |
| *Lutrogale perspicillata* | 73.74607 | 16.25429 |
| *Lutrogale perspicillata* | 73.4292 | 17.57937 |
| *Lutrogale perspicillata* | 78.55408 | 10.89751 |
| *Lutrogale perspicillata* | 76.51263 | 15.35811 |
| *Lutrogale perspicillata* | 79.3124 | 16.57597 |
| *Lutrogale perspicillata* | 81.75314 | 16.94266 |
| *Lutrogale perspicillata* | 74.69821 | 13.64074 |
| *Lutrogale perspicillata* | 80.51351 | 16.30733 |
| *Lutrogale perspicillata* | 79.68787 | 11.79463 |
| *Lutrogale perspicillata* | 75.61817 | 24.99086 |
| *Lutrogale perspicillata* | 80.60193 | 16.50676 |
| *Lutrogale perspicillata* | 80.91226 | 15.92887 |
| *Lutrogale perspicillata* | 76.67109 | 15.44993 |
| *Lutrogale perspicillata* | 73.87531 | 15.53166 |
| *Lutrogale perspicillata* | 73.87059 | 15.50961 |
| *Lutrogale perspicillata* | 74.1437 | 15.24385 |
| *Lutrogale perspicillata* | 77.67286 | 11.96067 |
| *Lutrogale perspicillata* | 72.92091 | 21.27815 |
| *Lutrogale perspicillata* | 73.12677 | 21.01922 |
| *Lutrogale perspicillata* | 73.75992 | 21.83024 |
| *Lutrogale perspicillata* | 73.64687 | 21.88559 |
| *Lutrogale perspicillata* | 73.69014 | 21.22503 |
| *Lutrogale perspicillata* | 72.80779 | 21.61513 |
| *Lutrogale perspicillata* | 72.68081 | 21.43948 |
| *Lutrogale perspicillata* | 73.84886 | 23.33732 |
| *Lutrogale perspicillata* | 77.52372 | 12.84496 |
| *Lutrogale perspicillata* | 81.73309 | 17.06995 |
| *Lutrogale perspicillata* | 82.29848 | 16.82645 |
| *Lutrogale perspicillata* | 84.23518 | 23.68122 |
| *Lutrogale perspicillata* | 76.27939 | 10.91624 |
| *Lutrogale perspicillata* | 75.93907 | 12.44291 |
| *Lutrogale perspicillata* | 75.90589 | 12.35483 |
| *Lutrogale perspicillata* | 73.95556 | 15.48748 |
| *Lutrogale perspicillata* | 73.84884 | 15.52022 |
| *Lutrogale perspicillata* | 73.87668 | 15.59469 |
| *Lutrogale perspicillata* | 76.41279 | 9.598556 |
| *Lutrogale perspicillata* | 76.39406 | 9.662617 |
| *Lutrogale perspicillata* | 75.57871 | 24.90095 |
| *Lutrogale perspicillata* | 75.58362 | 24.91714 |
| *Lutrogale perspicillata* | 75.82404 | 25.17414 |
| *Lutrogale perspicillata* | 75.67641 | 25.03615 |
| *Lutrogale perspicillata* | 92.33761 | 26.54345 |
| *Lutrogale perspicillata* | 75.13168 | 31.33703 |
| *Lutrogale perspicillata* | 76.09349 | 10.82681 |
| *Lutrogale perspicillata* | 77.20465 | 9.526981 |
| *Lutrogale perspicillata* | 77.40638 | 8.503675 |
| *Lutrogale perspicillata* | 90.97093 | 26.74466 |
| *Lutrogale perspicillata* | 91.71711 | 27.69783 |
| *Lutrogale perspicillata* | 86.86568 | 20.71073 |
| *Lutrogale perspicillata* | 86.85226 | 20.72229 |
| *Lutrogale perspicillata* | 86.87503 | 20.72386 |
| *Lutrogale perspicillata* | 86.86912 | 20.70191 |
| *Lutrogale perspicillata* | 86.8524 | 20.70524 |
| *Lutrogale perspicillata* | 86.87503 | 20.71344 |
| *Lutrogale perspicillata* | 79.76058 | 11.38389 |
| *Lutrogale perspicillata* | 79.76874 | 11.38907 |
| *Lutrogale perspicillata* | 79.76156 | 11.39467 |
| *Lutrogale perspicillata* | 73.2279 | 22.63704 |
| *Lutrogale perspicillata* | 79.72942 | 11.91744 |
| *Lutrogale perspicillata* | 79.73659 | 11.9112 |
| *Lutrogale perspicillata* | 86.18003 | 21.89572 |
| *Lutrogale perspicillata* | 86.16801 | 21.84589 |
| *Lutrogale perspicillata* | 86.19673 | 21.69223 |
| *Lutrogale perspicillata* | 86.21921 | 21.64637 |
| *Lutrogale perspicillata* | 86.31916 | 21.73978 |
| *Lutrogale perspicillata* | 71.97274 | 22.0416 |
| *Lutrogale perspicillata* | 72.73397 | 21.12736 |
| *Lutrogale perspicillata* | 72.70325 | 21.22011 |
| *Lutrogale perspicillata* | 72.68544 | 21.10014 |
| *Lutrogale perspicillata* | 88.9393 | 21.99255 |
| *Lutrogale perspicillata* | 88.75645 | 21.76396 |
| *Lutrogale perspicillata* | 88.9713 | 21.88303 |
